# Both male and female identity influence variation in male signalling effort

**DOI:** 10.1186/1471-2148-11-233

**Published:** 2011-08-09

**Authors:** Topi K Lehtonen, P Andreas Svensson, Bob BM Wong

**Affiliations:** 1 School of Biological Sciences, Monash University, Victoria 3800, Australia; 2 Section of Ecology, Department of Biology, 20014 University of Turku, Finland; 3 School of Life and Environmental Sciences, Deakin University, Geelong, Victoria 3220, Australia

## Abstract

**Background:**

Male sexual displays play an important role in sexual selection by affecting reproductive success. However, for such displays to be useful for female mate choice, courtship should vary more among than within individual males. In this regard, a potentially important source of within male variation is adjustment of male courtship effort in response to female traits. Accordingly, we set out to dissect sources of variation in male courtship effort in a fish, the desert goby (*Chlamydogobius eremius*). We did so by designing an experiment that allowed simultaneous estimation of within and between male variation in courtship, while also assessing the importance of the males and females as sources of courtship variation.

**Results:**

Although males adjusted their courtship depending on the identity of the female (a potentially important source of within-male variation), among-male differences were considerably greater. In addition, male courtship effort towards a pair of females was highly repeatable over a short time frame.

**Conclusion:**

Despite the plasticity in male courtship effort, courtship displays had the potential to reliably convey information about the male to mate-searching females. Our experiment therefore underscores the importance of addressing the different sources contributing to variation in the expression of sexually-selected traits.

## Background

Males often rely on elaborate sexual displays to attract females. Such displays can reveal important information about the quality or motivation of the signaller [1,2]. Courtship displays are therefore commonly used by females as cues when selecting a mate and, in so doing, affect the regime of sexual selection [3,4]. Considerable research attention has been given to understanding among-male variation in signal expression due to its potential in influencing male mating opportunities and reproductive success. Less well studied - but just as important - is the variation in signal intensity that can occur within individuals [5,6] due to behavioural plasticity [e.g. [7]]. Such variation can arise, for example, in response to life-history trade-offs between present and future signalling effort, as in *Drosophila *[8] and three-spined sticklebacks, *Gasterosteus aculeatus *[6,9], or between different components of male reproductive investment (e.g. mate attraction versus parental care), as in collared flycatchers, *Ficedula albicollis *[10,11]. Males might also be expected to adjust their courtship effort strategically to maximise their reproductive payoffs, especially if mating costs are high, if females vary greatly in reproductive value, and if there is a good chance of attracting high quality mates in the future [12]. In general, both among- and within-individual variation in courtship intensity can affect the evolutionary potential of sexual selection by contributing to variance in reproductive success [11].

One useful approach for investigating sexual displays at the population level is to measure the display intensity of several individuals more than once [13]. Such data can then be used to estimate the fraction of variation in display behaviour that is due to differences among individuals, that is, the 'repeatability' of the behaviour [14,15]. Repeatability has been widely used to understand evolutionary processes (e.g. heritability), with high repeatability values indicating high consistency within, and large differences among, individuals [13-15]. Low values, by contrast, would indicate the opposite pattern. Quite remarkably, while many studies of sexual selection have assessed repeatability of male courtship effort [13], very few have dissected different sources of variation in male courtship and compared them directly, despite the potential value of such comparisons [see [16]].

**Figure 1:**
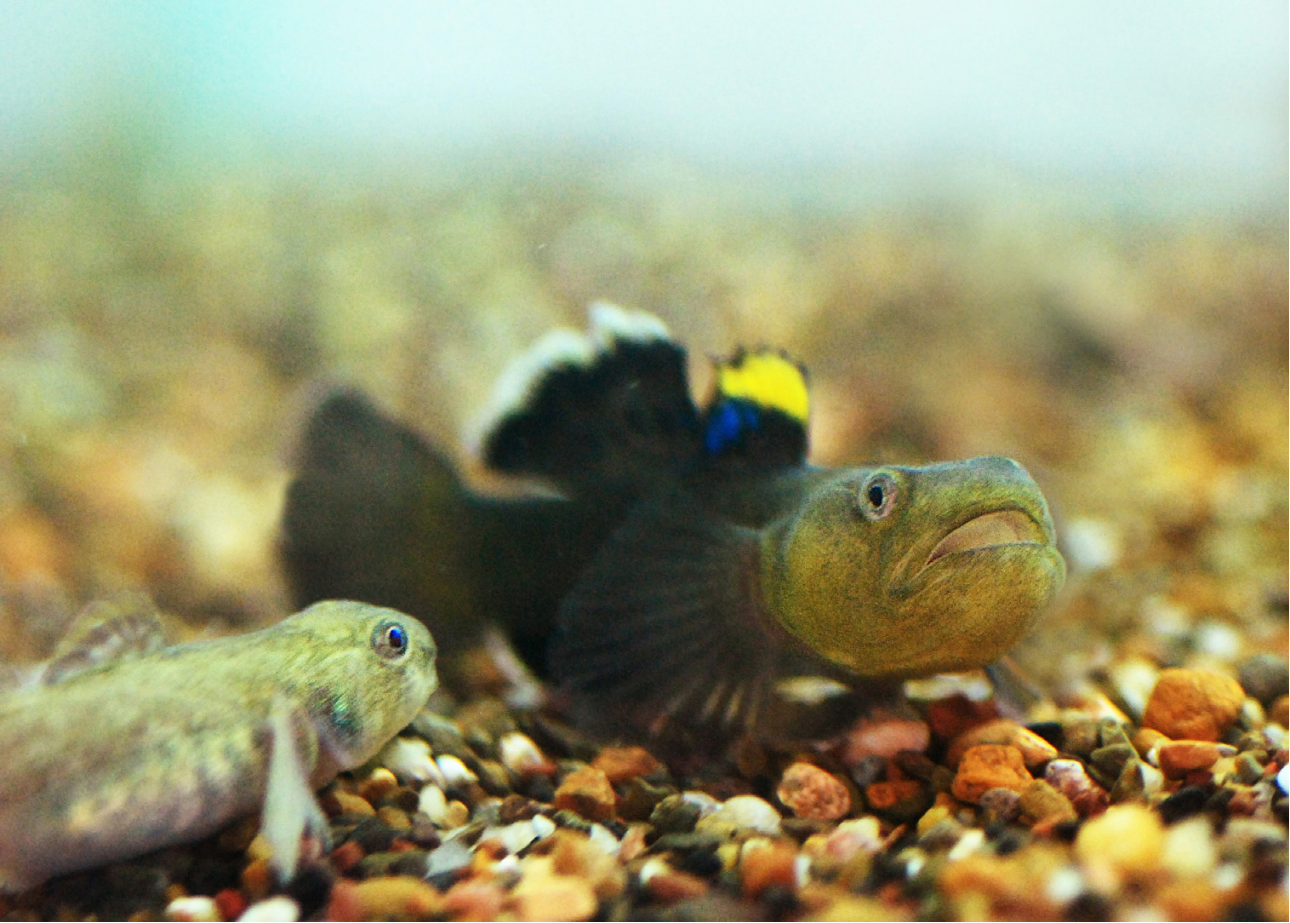
**Male desert goby (*Chlamydogobius eremius*) courting a female**.

The desert goby, *Chlamydogobius eremius*, is a freshwater fish endemic to the Lake Eyre drainage basin in Central Australia. This small (≤ 8 cm), sexually-dimorphic species (Figure 1) is locally abundant throughout its range where it inhabits both permanent and temporary bodies of water, from spring-fed pools to ephemeral desert streams. Male desert gobies establish nests under rock crevices and rely on conspicuous displays to attract passing females for mating. The courtship displays involve the raising of the colourful dorsal and anal fins coupled with occasional jerky body movements (Figure 1, additional file 1, [17]). Previously, males were shown to adjust their courtship effort strategically by courting larger females more intensely [17]. The aim of the current study was to simultaneously assess potential sources of individual variation in male courtship effort. Specifically, if male courtship is to be a potentially useful sexual signal, we predict that, for a given point in time, variation in signalling effort observed among males should be greater relative to the variation attributed to female identity, an important source of within-male variation [17]. If, in turn, differences among males in courtship were small compared to each male's tendency to adjust his courtship effort, or relative to short term within-male variability due to other reasons, male courtship effort would be less reliable as a signal to females. We would also expect courtship effort to be repeatable (*sensu *Becker [14]). Accordingly, we designed an experiment that could compare the different sources of variation in male courtship (male identity, female identity, and unidentified sources) directly, as well as assess the repeatability of courtship effort (by also allowing us to partition the total variation into within-male and among-male sources).

## Methods

### Fish collection and housing

Experimental trials took place from January to May 2009. Desert gobies were collected as juveniles from waterholes and springs west of Lake Eyre in South Australia using dip and seine nets. During a two-day surface transport to the laboratory, fish were kept in insulated 50-litre plastic tubs (coolers), filled with water to a depth of 30 cm and provided with constant motorised aeration. Back in the laboratory, fish were housed in 80 - 300 L aquaria and were separated by sex after maturation. Aquaria were maintained at a temperature of 23 - 26°C, a salinity of 5 - 7‰ and on a 12 h light:dark cycle. All fish were fed 1 - 2 times a day *ad libitum *on a diet of commercially prepared pellets and frozen brine shrimp (*Artemia*). After completion of the study, fish were retained as stock for unrelated research.

### Measurement of courtship effort and fish size

Male desert gobies were introduced into individual compartments (measuring 18 × 25 cm, with water depth of approximately 20 cm) containing a halved clay flowerpot as a nest (diameter = 6.5 cm, length = 6.5 cm). The entrance of the nest was positioned to face a small (6 × 25 cm), adjoining female compartment separated by a clear Perspex divider (see also Figure 1B in [17]). The bottom of both compartments was covered with a 3 cm layer of sand.

After the male (total length ± standard error: 65 ± 1.4 mm, *N *= 40) was introduced into his compartment, he was given three days to acclimate before we introduced a stimulus female (53 ± 1.1 mm, *N *= 40) into the adjacent compartment. To standardise female reproductive state, only ripe females were used, as determined by their distended bellies. Data on fish behaviour was collected following previously published methods [17,18]. Tanks were brightly lit from above, with the observer seated in the dark away from the tank to prevent disturbance to the fish. Three minutes after the introduction of the stimulus female, we measured the amount of courtship directed by the male towards the female. This was achieved by conducting spot samples every 10 seconds over a 10 minute period. During each spot sample, a male was recorded as courting the female if he was within 5 cm of her compartment, with his body oriented unambiguously towards her whilst engaged in courtship behaviour (i.e. fin displays) [18]. At the end of the trial, we tallied the total number of times the male was courting the female as a measure of his signalling effort.

Male courtship effort was measured over two subsequent days so that two different males were each presented with the same pair of stimulus females. This combination of four fish is hereafter referred to as an experimental unit (*N *= 20). During the first day, both males were tested once with each of the two stimulus females within their experimental unit. Hence, during the first day, each fish performed in two trials. There was at least 45 minutes between each trial and the order of trials was randomised. The same procedure (with a new randomisation of the order of trials) was repeated on the following day.

Our experimental design provides more detailed information than the coefficient of intra-class variation (i.e. 'repeatability') alone and, importantly, allows testing of multiple hypotheses within a single experimental set up. Specifically, in order to estimate the variance components of male courtship effort (in terms of the contributions of male identity and female identity to the total variance in courtship intensity), we analysed the courtship data with linear mixed effects models, using the 'lme4' package in the R 2.10.1 software [19]. A model was fitted using day ('1' or '2') as a fixed factor and female ID and male ID as random factors (random intercepts, common slopes). The statistical significance of random and fixed effects was assessed by comparing a full model with reduced models not containing the factor of interest, using log-likelihood tests [20]. When comparing models differing in their fixed factors, maximum likelihood was used to fit the models. In all other cases, restricted maximum likelihood was used.

Total lengths were measured by photographing each fish after the experiment in a container with 3 cm of water and a piece of grid paper as a scale. We used an Olympus C-5060 digital camera for photographs and Image Tool 3.00 (The University of Texas Health Science Institute, San Antonio, TX) software for the image analysis. The size difference between the two females within each experimental unit ranged from zero to moderate (average length difference between the two females: 4.9 ± 1.3 mm). Similarly, large size differences between each of the two males were avoided (average length difference between the two males: 5.4 ± 0.75 mm). We tested whether differences in body size, as measured over the whole data-set, predicted patterns in male courtship effort by regressions of courtship effort as a function of body size, both for females and males.

One male did not engage in any courtship behaviour and remained inactive over the whole period of the experiment, and was therefore excluded from analyses.

### Repeatability of courtship effort

In order to get an estimate for repeatability of male courtship effort (and its standard error and 95% confidence intervals), we analysed the total amount of courtship each male performed during days 1 and 2, following the procedure of Becker [14]. Specifically, a one-way ANOVA was used to partition the total variance of courtship intensity into the variance among males (*S*^2^_A_) and the variance within males (*S*^2^). The ratio of the variance among males (*S*^2^_A_) to the total phenotypic variance (*S*^2 ^+ *S*^2^_A_) then gives an estimate of repeatability, also called 'the coefficient of intra-class correlation' *sensu *Lessells & Boag [15].

### Ethical note

This study complies with all the relevant Federal and State laws of Australia, adheres to the ASAB/ABS guidelines for the use of animals in research, and was conducted under ethics permit 'BSCI/2007/12' from the Biological Sciences Animal Ethics Committee of Monash University.

## Results

**Figure 2:**
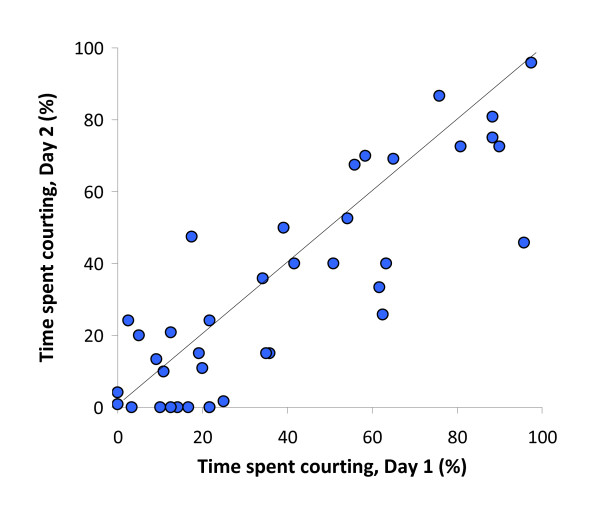
**Time spent courting by males on days 1 and 2**. Each data point (*N *= 39) represents the percentage of time an individual male spent courting (as averaged for the two stimulus females, and calculated from spot samples of male behaviour). The line indicates identical courtship effort on the two days.

**Figure 3:**
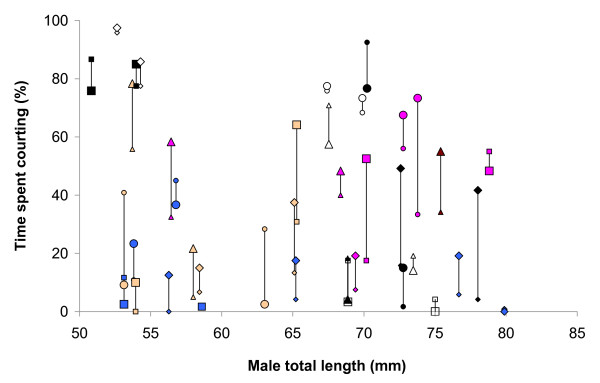
**Variation in male courtship effort, with males (*N *= 39) ordered according to their body size**. The two vertically attached symbols represent courtship effort towards the two stimulus females within a single focal male's 'experimental unit'. The larger of the two females is indicated with a larger symbol and the smaller female with a smaller symbol. The two males within each unit (i.e. the two males that courted the same pair of females) are identified by a shared combination of symbol colour and type.

**Figure 4:**
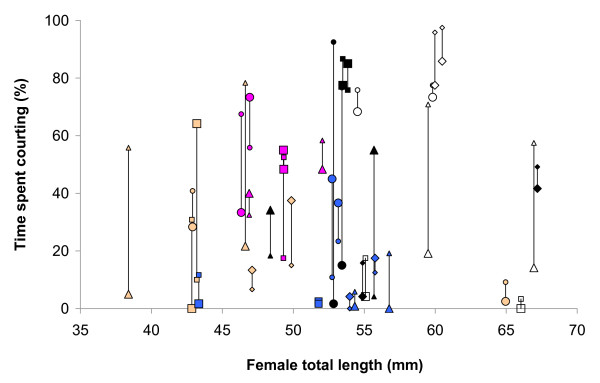
**Variation in male courtship effort due to female identity**. The two vertically attached symbols represent courtship efforts by the two males that courted the same stimulus female. The larger of these two males is indicated with a larger symbol and the smaller male with a smaller symbol. The two females within each experimental unit (i.e. the two females that were courted by the same two males) are identified by a shared combination of symbol colour and type.

The linear mixed effects model revealed a significant effect of 'day' on male courtship (with 15.9 ± 6.4% fewer displays on the second day: model 1 vs. model 3, *χ*^2 ^= 6.17, *df *= 1, *P *= 0.013; Figure 2; Table 1). There was also a significant contribution of male ID (model 1 vs. model 4, *χ*^2 ^= 60.6, *df *= 1, *P *< 0.001; Figure 3; Table 1) and of female ID (model 1 vs. model 2, *χ*^2 ^= 4.45, *df *= 1, *P *= 0.035; Figure 4; Table 1) to the fit of the model. 'Male ID' explained 69.0% of the total variance in male courtship, while 'female ID' (as a source of within-male variation under special interest) explained 6.0% and 'day' explained 1.6%. The remaining 23.4% was left unexplained by the model (residual variance).

**Table 1:** The degree of model fit (AIC) in linear mixed effects models of courtship effort in male desert gobies

Model	Fixed factors	Random factors	AIC	ΔAIC
1	Day	Male ID, Female ID	1253	0
2	Day	Male ID	1255	2
3		Male ID, Female ID	1260	7
4	Day	Female ID	1311	58

The ΔAIC is the difference in AIC values compared to the estimated best model (at the top of the table).

There was no significant relationship between the size of males and their total courtship effort as expressed over the two days (linear regression, *F*_1,37 _= 1.198, *R^2 ^*= 0.031, *P *= 0.28; Figure 3). Similarly, there was no significant relationship between the size of females and the amount of courtship that males directed toward them (linear regression, *F*_1,38 _= 0.680, *R^2 ^*= 0.018, *P *= 0.42; Figure 4). Male courtship effort, averaged towards the two females, was highly and significantly repeatable (the associated *F *= 11.30, repeatability: *r *= 0.837, *SE *= 0.049, 95% confidence interval: 0.713 - 0.911, *N *= 39 males; Figure 2).

## Discussion

Our results show that desert goby males exhibit considerable among-individual variation in courtship effort. Moreover, over a short time frame (here one day), these individual differences were also highly consistent. This is important because such consistency provides a potential for female choice (or, indeed, other forms of selection) to work on traits that affect courtship rate [21]. In this regard, both the level of heritable variation and the degree of condition dependence are potentially relevant [11]. Substantial individual variation in male courtship effort seems to be widespread among taxa (e.g. fish: [22]; frogs: [23]; arthropods: [24]; lizards: [25]) although this variation is usually not as pronounced as what the current study has shown for desert gobies (for points of comparison, see [13]). The extensive variation among desert goby males is unlikely to be due to differences in body condition (see [26-29]) as all males were fed *ad libitum*. Moreover, we did not find any effect of male body size on courtship.

Within-male differences in the expression of sexual signals can also be important. For example, previous studies have found that environmental factors [23,30] and variation in female reproductive state [25,31] can be important sources of within-male variation in male signalling intensity. Indeed, in desert gobies, males are known to adjust their courtship effort strategically towards females of different reproductive value (based on dichotomous assessment of female body size: [17,18]). In the current study, however, controlled laboratory conditions minimised environmental variation, while the experimental design controlled for order effects. We also deliberately avoided large size differences between the two females seen by each male, as well as variation in their reproductive state (all females were ripe with eggs). Consequently, female size differences did not predict male courtship effort. We nevertheless found a significant effect of female identity on the level of male courtship effort, indicating that the males nonetheless perceived some difference between the females. These between-female differences could, for example, be related to colouration or behaviour (especially responsiveness), and provide an interesting avenue for future research. Indeed, female identity, as a source of within-individual variation in male courtship effort, may be especially important because plasticity in the expression of sexual signals can influence both signal reliability [32] and the power of sexual selection [11].

Even though desert goby males are capable of strategically adjusting their courtship (both in this study and according to female size in [17,18]), the current results indicate that courtship effort of a male is consistent when he experiences a similar situation on two adjacent days. Indeed, the differences among males were more pronounced than short term variation within males (with 69% of variation in courtship intensity being explained by male ID versus 6% explained by female ID). The consistency in male courtship effort was also demonstrated by a high estimate for its repeatability (*r *= 0.837). Thus, even though a male desert goby can strategically adjust its courtship effort, his displays were highly consistent when re-encountering the same females.

If among-male differences in courtship are more important than variation within males, females have the potential to reliably assess males based on courtship intensity. Indeed, in many species, female mating decisions seem to be biased towards males that exhibit high rates of courtship [3,33-35], and among-male differences in courtship effort often translate into differences in male mating success. However, in species where female traits affect male courtship intensity, we might expect courtship to be less informative of male attributes (such as body condition, parental ability or genetic quality) than in systems where courtship displays are less flexible. Furthermore, even when courtship effort plays a role in mate choice, the relationship between courtship intensity and female preference is not always clear-cut [36-38]. Females, for instance, could be using a suite of different cues in mate choice [39,40] or they may be inconsistent in their choice of potential mating partners [13,21]. For example, in the sand goby, a species with a mating system very similar to that of desert gobies, females exhibit individually varying mate preferences [16] that are dependent on social context [41] and also fluctuate over time [42].

## Conclusion

We found that among-male differences in courtship effort in desert gobies were more pronounced than differences due to female identity or other within-male sources. Thus, males were consistent in their investment in courtship displays when confronted with the same two females on adjacent days. The consistent among-male differences also resulted in a high repeatability estimate for courtship effort. Females, therefore, have the potential to reliably assess males based on intensity of their courtship. Our study underscores the importance of paying attention to each of the different sources of variation in the expression of male sexual displays, and we offer a robust experimental design for simultaneously estimating these parameters. Future investigations should consider the importance of male courtship for female mate choice relative to other sexually-selected traits that may also be important in guiding female mating decisions.

## Authors' contributions

TKL designed the study, conducted the experiment, participated in data analyses and wrote the bulk of the manuscript. PAS conducted the image measurements, performed most of the data analyses and participated in writing of the manuscript. BBMW guided the project, provided ideas throughout the study and contributed to writing of the manuscript. All authors read and approved the final manuscript.

## Supplementary Material

Additional file 1 **Desert goby courtship behaviour**. Video footage on courtship displays by a male desert goby (*Chlamydogobius eremius*). Click here for file
